# Special Issue: Animal Modeling in Cancer

**DOI:** 10.3390/genes11091009

**Published:** 2020-08-27

**Authors:** Vladimir Korinek

**Affiliations:** Institute of Molecular Genetics of the Czech Academy of Sciences, Videnska 1083, 142 20 Prague, Czech Republic; vladimir.korinek@img.cas.cz; Tel.: +420-241-063-146; Fax: +420-244-472-282

**Keywords:** cancer, mouse models, non-mouse models, gene editing, stem cells, solid tumors, hematologic malignancies

## Abstract

Recent advances in high-throughput sequencing techniques have significantly accelerated the development of personalized diagnostic tools and cancer treatments. However, a comparative analysis of experimental animals that share similar genetic, physiological, and behavioral traits with humans remains the basis for understanding the pathological mechanisms associated with human diseases, including cancer. The generation and characterization of suitable animal models mimicking tumor growth and progression thus represents an important “component” of tumor biology research. The presented Special Issue contains ten review articles, which, based on data obtained from various animal models, summarize a number of aspects of the tumor formation process that include gastrointestinal neoplasia, breast cancer, hematological malignancies, melanoma, and brain tumors. This Special Issue nicely illustrates how the study of suitable living models uncovers not only the fundamental molecular and cellular bases of neoplastic growth, but might also indicate approaches to efficient cancer treatments.

The use of animal models to study the process of cell transformation and tumor formation has become a routine experimental approach. Initially, various types of neoplasms were induced in the experimental animal by exogenous substances or radiation. These techniques were extended by genetic models. Such models were first obtained by selecting “random” phenotypes, and later also by targeted genome modifications. Additionally, xenotransplantation techniques for implanting tumor cells or tumor tissue fragments directly into animals’ bodies have been developed. Another technical improvement that accelerated and streamlined the preparation of animal models was based on the introduction of the method of conditional genetic modifications (especially the Cre/loxP system), which enabled tissue-specific and time-defined genetic changes. A breakthrough was brought by the introduction of programmable nuclease technology and the clustered regularly interspaced short palindromic repeats (CRISPR)/CAS9 system. The tumor process is associated with a number of genetic changes in the particular cell type, and these inventions have led to well-defined and sophisticated tumor models that resemble the complex pathological situations observed in human tumor tissue. This Special Issue has been dedicated to animal models used for cancer research and contains ten review articles. Not surprisingly, most of them are dedicated to rodents (especially mice and rats) as the main experimental model used in tumor biology.

Non-mammalian models are dominated by zebrafish (*Danio rerio*). The use of fish in biomedical research has its obvious advantages. These are, in particular, the relatively low costs of their breeding (related to a high reproductive capacity), extracorporeal development of embryos, and the possibility of performing genetic manipulations. The above advantages are somewhat complicated by the fact that during evolution, the zebrafish genome has undergone duplication, and its genes thus have more paralogs. The resulting redundancy or, conversely, species-specific variability of the original function of the studied gene might complicate the analysis and interpretation of the obtained results. On the other hand, the natural transparency of zebrafish embryos and the existence of a mutant strain called *casper*, whose individuals are transparent even in adulthood, making it possible to monitor the growth and distribution of tumor cells directly in a living organism. Another complication is the relatively small amount of commercially available antibodies that recognize fish antigens. However, this disadvantage can be very well compensated for by the use of transgenic “reporter” strains, a large number of which are currently available. The above-mentioned features, together with the fact that the development of the hematopoietic system has cellular bases and regulatory mechanisms very similar to those described in mammals, make zebrafish a unique model for the study of hematooncological diseases. This is well illustrated by examples of myeloproliferative neoplasms (MPN) [1] and acute myeloid leukemias (AML) [2]. A comprehensive summary of zebrafish as a model in tumor biology with a detailed presentation of various types of studied neoplasms and techniques is given in a review article by M. Hason and P. Bartunek [3].

Carcinoma of the colon and rectum (colorectal cancer (CRC)) represents one of the most frequently diagnosed neoplasia in developed countries. Therefore, it is also not surprising that the two articles included in the Special Issue are dedicated to mouse models of intestinal cancer. The majority of CRCs progress through the conventional adenoma–carcinoma pathway. In recent years, there have been considerable efforts to unify the classification of colorectal tumors. These efforts have led to the establishment of the system of four consensus molecular subtypes (CMSs) defined by multiple characteristics that include gene expression profiles, microsatellite instability, genomic DNA methylation status, and differences in the status of the immune response and various signaling pathways. In their review, M. Stastna and colleagues have presented a broad spectrum of mouse intestinal cancer models that display pathological changes in Wnt, Hippo, p53, epidermal growth factor (EGF), and transforming growth factor β (TGFβ) signaling. They have also described microsatellite instability models and models of chemically induced tumorigenesis. Importantly, the authors reflect the categorization of human CRC into the CMS groups and indicate a possible assignment of the described mouse model to the CMS group [4]. The mammalian gut is a complex organ consisting not only of cells intrinsic for the organism, but also containing vast amounts of bacteria. Importantly, microbial colonization of the gut is essential for the development and function of the immune system, proper digestion and acquisition of nutrients, and vitamin production. In a well-structured and interesting comprehensive article, A.A. Leystra and M.L. Clapper present the topic of the influence of intestinal microbiome compositions on the phenotypes of mouse models with colon cancer. The article further summarizes the factors that significantly affect the composition of the intestinal microbiome in experimental mice. Importantly, the authors also propose strategies that might help to evaluate the effect(s) of the differing microbiome composition on the output of experiments related to intestinal tumorigenesis [5].

The issue of intestinal neoplasias is related to the article of K. Chawengsaksophak, which is fully dedicated to the role of the caudal type homeobox 2 (*Cdx2*) gene in esophageal and gastrointestinal cancer and leukemias, as shown by the analysis of mice and zebrafish models. Cdx2 is an important factor in defining the positional identity of cells. Pathological situations associated with tissue damage and its subsequent regeneration may cause changes to the cell identity called metaplasia, or an abnormal localization of otherwise morphologically and cytologically normal tissue. Importantly, metaplasias represent a significant factor in the development of cancer. An aberrant expression of the *Cdx2* gene induces a shift in cellular identity towards more posterior types and, in animal models, it results in an intestinal type of metaplasia of the esophageal epithelium (so-called Barrett’s esophagus) and stomach. In contrast, a loss of the *Cdx2* gene in the colon induces neoplasias producing gastric markers. Finally, leukemia is one of the neoplasms for which Cdx2 (over)production is typical [6].

Two articles are dedicated to hematooncological models. L. Lanikova and colleagues thoroughly describe the genetic basis (the so-called mutation landscape) of MPN, which are represented by polycythemia vera, essential thrombocytosis and primary myelofibrosis. The authors introduce mouse and fish models with these disorders. They also provide examples of how the technology of induced pluripotent stem cells (iPSCs) has initiated a new era in human disease modeling. For example, iPS cells prepared from MPN allow for the reconstruction of the clonal hierarchy and investigation of the effects of oncogenic mutations at their endogenous settings [1]. The article by H. Skayneh and co-workers deals with zebrafish and rodent (rat, mouse) AML models. AML is a very common hematological neoplasia with a heterogeneous genetic basis, and suitable animal models are a key tool for the analysis of individual AML (sub)types. The authors also report the use of Drosophila as an invertebrate model to study the chromosomal translocation t (8:21) (q22; q22), which is very frequent in AML [2].

The article by V. Horak and colleagues focuses on animal models used in the study of melanoma. Melanoma is a very aggressive and deadly type of cancer, the incidence of which is increasing worldwide, especially in the Caucasian population. The introductory part comprehensively describes our current knowledge about the genetic changes and molecular mechanisms related to the origin and progression of the disease. A detailed description of animal models follows, which includes not only mouse models, but also dog and horse models, as well as three non-mammalian animal models (zebrafish, Xiphophorus and Drosophila). The most extensive part of the review is dedicated to the Melanoma-Bearing Libechov Minipig. The origin of this genetic model dates back to the late 1960s, and the knowledge about this model is thus very deep and extensive. The (mini)pig as a model for melanoma research has a number of advantages. The structure and distribution of melanocytes in the pig skin is more similar (unlike mouse skin) to the situation in the human skin. Moreover, the relatively long lifespan (12–18 years) allows the long-term monitoring of the experimental animals. Large animals also allow blood or tissue samples to be taken repeatedly during their life (and disease progression) of the experimental individuals [7].

Research over the last few decades has clearly shown that dysfunction of the evolutionarily conserved Wnt, Notch, and TGFβ signaling pathways may have critical consequences for cellular fate, often leading to cell transformation and tumorigenesis. The article by N. Baloghova and colleagues offers a new perspective on the regulation of these signaling pathways by the ubiquitin–proteasome (UPS) system. The authors summarized (in an “exhaustive” manner) the results obtained in the mouse models [8]. It should be emphasized that for therapy based on UPS function manipulation, a thorough understanding of the molecular mechanisms affecting the protein stability is essential. Additionally, a subsequent validation of this knowledge in living animal models is also required.

The last two review articles offer a somewhat different view of the use of animal models in tumor biology. R.A. Moorhead evaluates the possibility of whether the consumption of soy products or soy-derived isoflavones might prevent (or reduce) the risk of breast cancer. The author summarizes that while epidemiological studies conducted in Asian countries show that high levels of soy and soy product consumption are associated with a reduced risk of breast cancer, experiments using mouse or rat breast tumor models (with tumors induced by chemical carcinogens or ectopic oncogenes) have not been confirmatory [9]. Obviously, the negative outcomes of the experiments could be related to the overall experimental design; however, the main issue might be that rodents metabolize soy isoflavones differently than humans. The article by A. Michaelidesova and co-workers describes the basic characteristics of various types of brain tumors, radiotherapy techniques, and possible side effects of radiotherapy. For obvious reasons, it is difficult to study neurogenesis in the adult human brain, but postnatal neurogenesis has been studied in detail in rodents, as nicely summarized in this article—[10]. Here, again, it should be emphasized that the question of neurogenesis in the adult human brain is still controversial. Which brings me back to the idea that some caution is needed when generalizing the results obtained in animal models.

I thank all the authors and reviewers of the published articles and I wish the readers inspiring reading.
